# Risk factors for delayed postoperative hyponatremia in patients with non-functioning pituitary adenomas undergoing transsphenoidal surgery: A single-institution study

**DOI:** 10.3389/fneur.2022.945640

**Published:** 2022-07-19

**Authors:** Yinxing Huang, Meina Wang, Jianwu Wu, Kunzhe Lin, Shousen Wang, Fangfang Zhang

**Affiliations:** ^1^Department of Neurosurgery, Fuzong Clinical Medical College of Fujian Medical University, Fuzhou, China; ^2^Department of Neurosurgery, The 900th Hospital of Joint Logistic Support Force, PLA, Fuzhou, China; ^3^Department of Neurosurgery, Affiliated Fuzhou First Hospital of Fujian Medical University, Fuzhou, China; ^4^Department of Neurosurgery, 900th Hospital, Fuzong Clinical Medical College of Fujian Medical University, Fuzhou, China; ^5^Department of Endocrinology, Affiliated Fuzhou First Hospital of Fujian Medical University, Fuzhou, China

**Keywords:** delayed hyponatremia, transsphenoidal surgery, non-functional pituitary adenoma, hyperprolactinemia, pituitary stalk

## Abstract

**Purpose:**

We aimed to assess factors influencing the occurrence of delayed hyponatremia after transsphenoidal surgery (TSS) in patients with a non-functional pituitary adenoma (NFPA).

**Methods:**

We retrospectively collected the clinical data of patients who underwent TSS for NFPA between January 2016 and January 2021. The pituitary region was preoperatively scanned with 3.0 T magnetic resonance imaging. The risk factors for delayed postoperative hyponatremia for NFPA were identified by univariate and multivariable logistic regression analysis.

**Results:**

We selected 166 patients with NFPA who fulfilled the inclusion criteria. Delayed postoperative hyponatremia occurred in 28 patients and did not in 138. Multivariable logistic regression analyses demonstrated that higher odds of developing delayed postoperative hyponatremia were independently associated with larger craniocaudal dimension (OR = 1.128, *P* = 0.034), as well as preoperative hyperprolactinemia (OR = 2.618, *P* = 0.045) and larger preoperative pituitary stalk deviation angle (OR = 3.033, *P* = 0.022).

**Conclusion:**

We identified the independent risk factors for delayed hyponatremia after TSS for NFPA; these included preoperative hyperprolactinemia, craniocaudal diameter, and preoperative pituitary stalk deviation angle.

## Introduction

Pituitary adenoma is a common benign intracranial tumor, accounting for 10~15% of the intracranial tumors (1). According to the levels of hormone secretion, pituitary adenomas can be classified into functional pituitary adenomas and non-functional pituitary adenomas (NFPAs). NFPAs are usually diagnosed based on signs and symptoms associated with the effects of tumor mass (headache, visual disturbance, and sometimes pituitary apoplexy) because the clinical symptoms associated with increased hormones are usually absent, but they may also be found incidentally (2). Surgical resection remains the first-line treatment for NFPA (3).

Although a transcranial approach is possible, currently, the most common intervention for NFPA is transsphenoidal surgery (TSS). The transsphenoidal approach avoids traction on the brain and cranial nerves during tumor resection. This approach typically offers up to 90% improvement in preoperative symptoms such as those due to the mass effect, including headache and vision loss (3). TSS can achieve maximal tumor resection, relatively less surgical trauma, fewer postoperative complications, and shorter hospital stays. Patients without complications can be discharged with home care instructions within 2–3 days after TSS (4).

Nevertheless, delayed postoperative hyponatremia remains an important problem that must be investigated. Delayed postoperative hyponatremia refers to serum sodium levels of <135 mmol/L on or after postoperative day 3 (5). Studies have shown that delayed postoperative hyponatremia is the leading cause of unplanned readmission within 30 days of TSS (6, 7). Severe hyponatremia can lead to prolonged hospitalization and even life-threatening conditions (8). Therefore, predicting the incidence of delayed hyponatremia after TSS has been attracting the attention of many clinicians. However, currently, there is no consensus on the predictors that can serve as early indicators for clinicians to detect.

Observation of the morphological characteristics of tumors by magnetic resonance imaging (MRI) may help in predicting the occurrence of postoperative complications. Oh et al. found that the craniocaudal diameter of the tumor could help predict the occurrence of postoperative diabetes insipidus in NFPA (9). However, this indicator has not yet been reported to predict the occurrence of delayed hyponatremia after TSS for NFPA.

The aim of this study was to provide risk factors for the occurrence of delayed hyponatremia after TSS for NFPA through preoperative observation of MRI findings of the tumor and pituitary stalk, along with laboratory indicators.

## Methods

### Study cohort

The clinical data of patients who underwent TSS for NFPA in Fuzong Clinical Medical College of Fujian Medical University between January 2016 and January 2021 were collected retrospectively. Our inclusion criteria were as follows: a) patients with NFPA confirmed by clinical and pathological examination, b) TSS performed by the same surgeon with more than 10 years of surgical experience, c) pituitary adenoma resection for the first time, and d) complete clinical data available. We excluded those with a history of drug therapy or radiation therapy for NFPA and those with NFPA combined with other pituitary lesions, such as Rathke's cleft cysts. All patients participating in the study provided informed consent. The study protocol was approved by Fuzong Clinical Medical College of Fujian Medical University.

### Data collection

**Figure 1:**
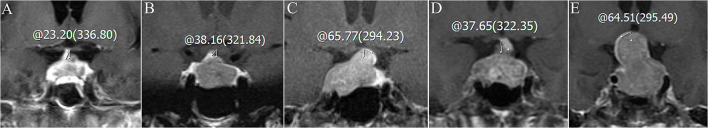
Coronal contrast-enhanced images of the pituitary stalk deviation angle before surgery. **(A)** The pituitary stalk deviates 23.20° (0° ≤ angle <30°) to the right (normonatremia group). **(B)** The pituitary stalk deviates 38.16° (30° ≤ angle <60°) to the right (normonatremia group). **(C)** The pituitary stalk deviates 65.77°(60° ≤ angle <90°) to the left (normonatremia group). **(D)** The pituitary stalk deviates 37.65°(30° ≤ angle <60°) to the left (delayed hyponatremia group). **(E)** The pituitary stalk deviates 64.51° (60° ≤ angle <90°) to the right (delayed hyponatremia group).

We collected data on patient demographics (sex and age), laboratory test results, and MRI scans. Laboratory tests included preoperative free triiodothyronine, free thyroxine, thyroid-stimulating hormone, cortisol, adrenocorticotropic hormone, prolactin, and growth hormone. After surgery, serum sodium level was measured daily until the patient was discharged. Delayed postoperative hyponatremia was defined as serum sodium levels of <135 mmol/L on or after postoperative day 3 (5). The degree of hyponatremia was evaluated as follows: mild (130–134 mEq/L), moderate (125–129 mEq/L), and severe (<125 mEq/L) hyponatremia. Hyperprolactinemia is defined as a plasma prolactin level of >18 ng/ml in men or 25 ng/ml in women (10). The pituitary region was scanned with 3.0 T MRI before surgery. The location of the tumor (major part in supra-sellar, intra-sellar or sphenoidal sinus) was observed. Measurement of transverse, craniocaudal, and anteroposterior dimensions were recorded using T1-enhanced images. Tumor volume was calculated using the simplified ellipsoid volume equation (10). The angle at which the pituitary stalk deviates from the midline is measured in the coronal plane and called the pituitary stalk deviation angle (11) (Figure 1). Knosp grades 0–2 were defined as non-invasive adenomas and grades 3–4 as invasive adenomas (12). Measurements of MRI parameters were collected independently by a neurosurgeon and radiologist, respectively, and the average values were calculated for statistical analysis. For two measured values have a difference of more than 10% between the neurosurgeon and the radiologist, a consensus was reached after discussion, and the measurement was re-measured again.

### Perioperative management

All patients were evaluated for pituitary function before operation. For those with hypofunction of the adrenal axis and hypothyroidism, physiological doses of glucocorticoid and levothyroxine were administered as a replacement therapy, if needed. The tumor was resected using the single-nostril transsphenoidal approach under a microscope, the sphenoid sinus was packed with an expanded sponge. After operation, intravenous fluids were administered to supplement physiological needs, and the patients were gradually transitioned to self-feeding. Fluid intake should be restricted in patients with delayed hyponatremia caused by syndrome of inappropriate secretion of antidiuretic hormone (SIADH). If cerebral salt wasting syndrome is the cause, then sodium salt and blood volume should be supplemented actively. The expandable sponge was removed from the sphenoid sinus cavity on postoperative day 4.

### Statistical analysis

Statistical analyses were performed with IBM SPSS Statistics for Windows, ver. 19 (IBM Corp., Armonk, NY, USA). Quantitative variables are expressed as mean ± standard deviation or median (interquartile range). Continuous variables were compared using standard Student's *t*-test and categorical variables using Pearson's chi-square test or Fisher's exact test. Non-parametric tests were applied to variables with nonnormal distribution. Logistic regression analysis was conducted to identify risk factors associated with delayed postoperative hyponatremia in NFPA. Univariate logistic regression was initially performed to identify variables of interest, and covariates with a *p*-value of <0.05 were incorporated into multivariable logistic regression models to identify independent risk factors for delayed postoperative hyponatremia. A *p*-value of <0.05 was considered statistically significant.

## Results

### Patient characteristics and groups

From January 2016 to January 2021, a total of 337 patients underwent TSS for pituitary adenomas. Among them, 166 patients (51 women and 115 men) fulfilled the inclusion criteria and were enrolled in the study. The overall mean age was 57.9 ± 5.6 years. The average time for onset of hyponatremia was 5.28 ± 1.71 days, and the average duration was 5.57 ± 1.34 days. Table 1 summarizes the clinical characteristics of the study participants. Delayed postoperative hyponatremia occurred in 28 patients (delayed hyponatremia group). Among them, four patients had mild hyponatremia, 14 had moderate hyponatremia, and 10 had severe hyponatremia. Moreover, 138 patients did not develop delayed postoperative hyponatremia (normonatremia group).

**Table 1:** Characteristics of 166 patients who underwent TSS for NFPA.

**Variable**	**No**.
Age, mean ± SD, years	57.9 ± 5.6
Sex	
Male	115
Female	51
Anteroposterior dimension, mm	17.4 ± 1.2
Transverse dimension, mm	26.5 ± 3.2
Craniocaudal dimension, mm	25.3 ± 2.3
Tumor volume, cm^3^	17.4 ± 1.2
Preoperative the pituitary stalk deviation angle (°)	41.5 ± 18.9
Invasiveness	
Yes	19
No	147
Histopathological types of NFPA	
Gonadotroph	122
Corticotroph (densely granulated)	3
Corticotroph (sparsely granulated)	2
Null-cell	36
Lactotroph (densely granulated)	2
Lactotroph (sparsely granulated)	1
Transcriptional factor of NFPA	
SF-1	122
T-Pit	5
Pit-1	3
None	36

*TSS, transsphenoidal surgery; NFPA, non-functional pituitary adenoma*.

### Univariate analysis of risk factors for delayed postoperative hyponatremia

Patient characteristics (found in Table 2) were compared between the delayed hyponatremia and normonatremia groups. Both groups had similar distribution of patient sex and age (*p* = 0.635 and *p* = 0.787, respectively). Compared with the normonatremia group, average transverse dimension (25.8 vs. 21.7 mm, *p* = 0.002), craniocaudal dimension (28.6 vs. 25.2 mm, *p* = 0.008), and tumor volume (6.7 vs. 4.6 mm, *p* = 0.005) were significantly greater in the delayed hyponatremia group. There were no differences in tumor invasiveness, tumor's location between the two groups. In addition, the delayed hyponatremia group showed a higher proportion of patients with hyperprolactinemia and large preoperative pituitary stalk deviation angle (*p* = 0.022 and *p* <0.001, respectively). Of the 63 hyperprolactinemia patients, 41 were Gonadotrophic, 2 were Corticotropic (densely granulated), 19 were Null-cell, and 1 was Lactotrophic (densely granulated).

**Table 2:** Univariate analysis of delayed postoperative hyponatremia.

**Factors**	**Delayed hyponatremia*****n*** = **28**	**Normonatremia** ***n*** = **138**	***P-value***
Age	52.0 (45.0, 60.7)	54.0 (46.0, 61.2)	0.635
Sex			0.787
Male	20	95	
Female	8	43	
Anteroposterior dimension, mm	19.1 (16.2, 23.4)	17.8 (14.7, 20.6)	0.067
Transverse dimension, mm	25.8 (21.1, 28.7)	21.7 (18.6, 24.3)	0.002
Craniocaudal dimension, mm	28.6 (23.3, 38.4)	25.2 (18.9, 29.8)	0.008
Tumor volume, cm^3^	6.7 (4.2, 13.1)	4.6 (2.7, 7.3)	0.005
The location of tumor (major part)			0.647
supra-sellar	21	92	
intra-sellar	7	45	
sphenoidal sinus	0	1	
Invasiveness			0.894
Yes	3	16	
No	25	122	
Preoperative hyperprolactinemia			0.022
Yes	16	47	
No	12	91	
Preoperative the pituitary stalk deviation angle			<0.001
0° ≤ angle <30°	0	29	
30° ≤ angle <60°	20	96	
60° ≤ angle <90°	8	13	
Preoperative serum sodium level, mEq/L	139.8 (137.2, 142.2)	140.3 (138.4, 142.0)	0.629
Preoperative prolactinemia, ng/ml	20.64 (13.05, 40.52)	12.93 (7.72, 26.72)	0.003
Preoperative FT3, pmol/L	3.8 (3.3, 4.4)	4.2 (3.7, 4.8)	0.040
Preoperative FT4, pmol/L	10.9 (8.0, 13.2)	13.5 (10.6, 15.8)	0.003
Preoperative TSH, μIU/ml	2.1 (1.0, 2.9)	1.5 (0.8, 2.4)	0.115
Preoperative cortisol, μg/dl	10.8 (2.6, 15.5)	12.9 (5.0, 17.2)	0.438
Preoperative ACTH, pg/ml	21.5 (7.1, 33.8)	23.8 (10.4, 34.5)	0.643
Preoperative GH, ng/ml	0.17 (0.06, 0.31)	0.21 (0.08, 0.48)	0.327
Time from day of surgery to day of discharge, days	10.0 (9.0, 12.0)	7.0 (6.0, 8.0)	<0.001

*FT3, Free triiodothyronine; FT4, free thyroxine; TSH, thyroid-stimulating hormone; ACTH, adrenocorticotropic hormone; GH, growth hormone. Values are median (interquartile range)*.

*Normal reference range for prolactinemia: 2.1–18 ng/ml (men), 2.8–25 ng/ml (women); Normal reference range for FT3: 3.5–6.5 pmol/L; Normal reference range for FT4: 11.5–22.7 pmol/L; Normal reference range for TSH: 0.55–4.78 μIU/ml; Normal reference range for cortisol: 4.3–22.4 μg/dl; Normal reference range for ACTH: 7.2–63.3 pg/ml; Normal reference range for GH: ≤ 1 ng/ml*.

We also found that preoperative free triiodothyronine and free thyroxine levels were significantly lower in the delayed hyponatremia group (*p* = 0.04 and *p* = 0.003, respectively). There was no difference in preoperative thyroid-stimulating hormone, preoperative cortisol, preoperative adrenocorticotropic hormone, and preoperative growth hormone levels between the groups. Further, the delayed hyponatremia group had longer hospital stays than the normonatremia group (*p* <0.001, Table 2).

### Multivariable analysis of risk factors for delayed postoperative hyponatremia

To determine which factors were independently associated with delayed postoperative hyponatremia, a multivariable logistic regression model was constructed (Table 3). Higher odds of developing delayed postoperative hyponatremia were independently associated with larger craniocaudal dimensions [odds ratio (OR) = 1.128, *p* = 0.034], preoperative hyperprolactinemia (OR = 2.618, *p* = 0.045), and a large preoperative pituitary stalk deviation angle (OR = 3.033, *p* = 0.022).

**Table 3:** Multivariate logistic regression analysis of risk of delayed postoperative hyponatremia.

**Factors**	**Odds ratio**	**95% CI**	***P-value***
Craniocaudal dimension	1.128	1.009, 1.261	0.034
Transverse dimension	1.111	0.958, 1.288	0.164
Tumor volume	1.000	1.000, 1.000	0.070
Preoperative hyperprolactinemia	2.618	1.020, 6.720	0.045
Preoperative the pituitary stalk deviation angle	3.033	1.176, 7.818	0.022
Preoperative FT3	0.743	0.409, 1.349	0.329
Preoperative FT4	0.864	0.739, 1.011	0.068

*FT3, Free triiodothyronine; FT4, free thyroxine*.

## Discussion

Delayed postoperative hyponatremia is one of the common complications after TSS for pituitary adenomas, with incidence of 23.4–26% (13–15). This study found that the incidence of delayed hyponatremia after TSS for NFPA was 16.9%, and we established independent risk factors for delayed hyponatremia after TSS for NFPA. These factors included craniocaudal dimension, preoperative hyperprolactinemia, and preoperative pituitary stalk deviation angle.

Studies have shown that the main cause of delayed hyponatremia after TSS for pituitary adenoma is SIADH (16). SIADH is the uncontrolled release of antidiuretic hormone due to mechanical damage caused by surgery to the pituitary stalk and posterior pituitary (17). It usually occurs 4–7 days after surgery (15). Another rare cause of delayed hyponatremia is cerebral salt wasting syndrome (4).

In fact, delayed hyponatremia after TSS has gradually gained the attention of clinicians. Currently, with the development of medical technology, some medical centers have greatly shortened the hospitalization time of patients undergoing TSS for NFPA, and patients can be discharged for home care within 2–3 days after surgery (4). However, this is associated with the problem of a higher rate of unplanned readmissions within 30 days of discharge. Among them, delayed postoperative hyponatremia is a major cause of unplanned readmissions (6). Therefore, identifying its reliable predictors may help guide postoperative management and improve medical safety for patients.

Several centers have reviewed these readmissions, but there is no consensus on the factors predicting delayed hyponatremia after TSS. Female sex and older age have been shown to be associated with delayed postoperative hyponatremia (5, 16, 18, 19). On the contrary, Rajaratnam et al. believed that men are at higher risk of delayed postoperative hyponatremia (14). Sorba et al. showed that younger age was an independent associated risk factor for predicting SIADH (20). In the current study, we did not find sex and age to be reliable predictors of delayed postoperative hyponatremia. In our center, it is routine for uncomplicated patients to be discharged around postoperative days 6–7. This is done for the following reasons: we remove the expandable sponge from the sphenoid sinus cavity on the 4th postoperative day and then continue to monitor the patient for epistaxis for 1–2 days; we are also monitoring the patients for the development of late-onset hyponatremia since most of the delayed hyponatremia occurs within 1 week of surgery. Therefore, the readmission rate in our center is very low.

Oh et al. reported that craniocaudal diameter can be used as a predictor of postoperative diabetes insipidus in NFPA (9). They suggest that when craniocaudal diameter is large enough, it can affect the hypothalamus to develop hypothalamic obesity, ultimately leading to a higher body mass index. In obese patients, because of the narrow nasal cavity, the operation is more difficult and may cause more damage to the hypothalamus or infundibulum (9). We found that craniocaudal diameter was an independent risk factor for delayed hyponatremia after TSS for NFPA. The aforementioned notions may explain the increased risk of delayed hyponatremia with a larger craniocaudal diameter. However, we believe that the theory based on the sinking of the sellar diaphragm is a better explanation. Most pituitary adenomas grow by pushing the sellar diaphragm, which sinks with surgical resection of the tumor (21). Recent reports suggest that the subsidence of the sellar diaphragm leads to pituitary stalk injury, which in turn leads to hyponatremia after TSS for pituitary adenoma (22). We believe that the larger the craniocaudal diameter, the more obvious the subsidence of the sellar diaphragm after tumor resection, and the greater the probability of delayed postoperative hyponatremia.

Our study demonstrates that the preoperative pituitary stalk deviation angle can be used to predict the occurrence of delayed postoperative hyponatremia. The pituitary adenoma can push the pituitary stalk as it grows upwards, causing the pituitary stalk to shift (11). We found that the preoperative pituitary stalk deviation angle was significantly greater in the delayed hyponatremia group than in the normonatremia group. The larger the deviation angle of the pituitary stalk, the more obvious the upward growth trend of the pituitary adenoma, which to some extent reflected the larger tumor volume in the suprasellar part. After the tumor is removed, the pituitary stalk can drastically change its original position or shape, resulting in damage to the pituitary stalk.

At last, our study also found that preoperative serum hyperprolactinemia was an independent risk factor for delayed hyponatremia after TSS for NFPA. In patients with pituitary adenomas, hyperprolactinemia is seen in addition to prolactinomas and can also occur in NFPA (10). Mechanical compression of the pituitary stalk by NFPA leads to the so-called “stalk effect” or “pituitary stalk compression syndrome.” This tumor-mediated physical compaction results in reduced dopamine (prolactin-inhibiting factor) release and a commensurate increase in prolactin output (23). Secondary hyperprolactinemia due to stalk effect may cause mild or moderate elevation of serum prolactin levels, but severe elevation is unlikely (24). For patients with NFPA and preoperative hyperprolactinemia, the possible cause of delayed postoperative hyponatremia can be the close association of the tumor to the pituitary stalk, which is more likely to be disturbed or damaged when the tumor is removed. Voglis et al. (25) built a machine learning model to predict hyponatremia after TSS and found that preoperative serum prolactin level is a significant predictive variable in the trained boosted generalized linear model. However, in addition to NFPA, they also incorporated growth hormone-secreting adenomas, prolactinomas, and others. Since the preoperative prolactin level of patients with prolactinoma is much higher than that in patients with NFPA, it is difficult to explain the cause of delayed hyponatremia through a simple “stalk effect.” However, only those with NFPA were included in our study, thus allowing the samples to be more homogeneous for a more robust study.

To the best of our knowledge, this is the first study to establish for the predictive value of preoperative serum prolactin levels in predicting delayed hyponatremia after TSS for NFPA. At the same time, through preoperative 3.0 T MRI evaluation, we found that the larger the craniocaudal diameter and preoperative pituitary stalk deviation angle, the higher the risk of delayed postoperative hyponatremia.

This study has its limitations. As this was a retrospective analysis, the sample size was relatively small. In addition, we only used preoperative indicators to predict the occurrence of delayed postoperative hyponatremia.

## Conclusions

We identified the independent risk factors for delayed hyponatremia after TSS for NFPA, including preoperative hyperprolactinemia, craniocaudal diameter, and preoperative pituitary stalk deviation angle. Preoperative evaluation of these factors can help physicians predict the patients who may develop delayed postoperative hyponatremia in the near future and ultimately use this information to manage these high-risk patients in a targeted, personalized manner.

## Data availability statement

The original contributions presented in the study are included in the article/supplementary material, further inquiries can be directed to the corresponding authors.

## Ethics statement

The studies involving human participants were reviewed and approved by Fuzong Clinical Medical College of Fujian Medical University. The patients/participants provided their written informed consent to participate in this study.

## Author contributions

YH, MW, and JW: data curation and writing original draft. KL: data retrieval and statistics. SW and FZ: designed the study and revised the manuscript. All authors reviewed the manuscript. All authors contributed to the article and approved the submitted version.

## Funding

This work was supported by General project of Natural Science Foundation of Fujian Province (2019J01530 and 2020J011185), Health young and middle-aged key Talents Training Program of Fujian Province (2020GGB045), and The 900th Hospital Clinical Application Research Special Project (2020L11).

## Conflict of interest

The authors declare that the research was conducted in the absence of any commercial or financial relationships that could be construed as a potential conflict of interest.

## Publisher's note

All claims expressed in this article are solely those of the authors and do not necessarily represent those of their affiliated organizations, or those of the publisher, the editors and the reviewers. Any product that may be evaluated in this article, or claim that may be made by its manufacturer, is not guaranteed or endorsed by the publisher.
